# Supplementary material for: Single Cell Transcriptomic and Chromatin Profiles Suggest Layer Vb Is the Only Layer With Shared Excitatory Cell Types in the Medial and Lateral Entorhinal Cortex
Source: Front Neural Circuits. 2022 Jan 26;15:806154. doi: 10.3389/fncir.2021.806154 (PMC8826650; doi:10.3389/fncir.2021.806154)
Supplement: Supplementary file 14 [file Data_Sheet_1.ZIP › 12/knownResults.html]

12 - Homer Known Motif Enrichment Results


# Homer Known Motif Enrichment Results (12)

Homer *de novo* Motif Results  
Gene Ontology Enrichment Results  
Known Motif Enrichment Results (txt file)  
Total Target Sequences = 12889, Total Background Sequences = 36449

|  |  |  |  |  |  |  |  |  |  |  |  |
| --- | --- | --- | --- | --- | --- | --- | --- | --- | --- | --- | --- |
| Rank | Motif | Name | P-value | log P-pvalue | q-value (Benjamini) | # Target Sequences with Motif | % of Targets Sequences with Motif | # Background Sequences with Motif | % of Background Sequences with Motif | Motif File | SVG |
| 1 | A G T C G A C T C A G T A C T G C T A G T G A C G C T A A T G C G C A T A T C G C G A T A C T G G A T C G T A C G T C A C T G A | NF1(CTF)/LNCAP-NF1-ChIP-Seq(Unpublished)/Homer | 1e-222 | -5.116e+02 | 0.0000 | 1722.0 | 13.36% | 2103.1 | 5.77% | motif file (matrix) | svg |
| 2 | C G T A C T A G T C A G T C A G A G T C A T G C A G T C G C A T A G C T A C G T A T C G C G A T | Sox9(HMG)/Limb-SOX9-ChIP-Seq(GSE73225)/Homer | 1e-179 | -4.125e+02 | 0.0000 | 2642.0 | 20.50% | 4254.2 | 11.68% | motif file (matrix) | svg |
| 3 | A G C T A C G T A C T G A T G C A G T C C G T A C T G A T A C G | NF1-halfsite(CTF)/LNCaP-NF1-ChIP-Seq(Unpublished)/Homer | 1e-155 | -3.575e+02 | 0.0000 | 4788.0 | 37.16% | 9633.7 | 26.45% | motif file (matrix) | svg |
| 4 | A G T C C G A T A C T G A T C G T G A C G C T A C A T G A T C G T G A C C G A T A C T G T A G C G T A C G T C A | Tlx?(NR)/NPC-H3K4me1-ChIP-Seq(GSE16256)/Homer | 1e-116 | -2.672e+02 | 0.0000 | 1610.0 | 12.50% | 2493.7 | 6.85% | motif file (matrix) | svg |
| 5 | A T G C A G T C G C A T A G C T A C G T T C A G C G A T A G C T G A T C A T C G | Sox10(HMG)/SciaticNerve-Sox3-ChIP-Seq(GSE35132)/Homer | 1e-112 | -2.585e+02 | 0.0000 | 3832.0 | 29.74% | 7743.8 | 21.26% | motif file (matrix) | svg |
| 6 | C G A T T G C A T G C A G A T C C G T A A C T G T G A C G A C T C A T G A C T G | Tcf21(bHLH)/ArterySmoothMuscle-Tcf21-ChIP-Seq(GSE61369)/Homer | 1e-110 | -2.538e+02 | 0.0000 | 2985.0 | 23.17% | 5684.4 | 15.61% | motif file (matrix) | svg |
| 7 | G T C A T G C A G C T A A G T C C G T A A C T G T G A C G C A T T C A G C A G T | Ap4(bHLH)/AML-Tfap4-ChIP-Seq(GSE45738)/Homer | 1e-105 | -2.425e+02 | 0.0000 | 3578.0 | 27.77% | 7197.6 | 19.76% | motif file (matrix) | svg |
| 8 | T C G A A G T C C G T A A T C G A T G C C G A T A C T G A G T C A G C T A C T G | Tcf12(bHLH)/GM12878-Tcf12-ChIP-Seq(GSE32465)/Homer | 1e-100 | -2.311e+02 | 0.0000 | 3011.0 | 23.37% | 5862.5 | 16.10% | motif file (matrix) | svg |
| 9 | C T A G T C G A T G A C A G T C C G T A A C T G G T A C A C G T A C T G A C T G | BHLHA15(bHLH)/NIH3T3-BHLHB8.HA-ChIP-Seq(GSE119782)/Homer | 1e-88 | -2.044e+02 | 0.0000 | 3779.0 | 29.33% | 7930.7 | 21.78% | motif file (matrix) | svg |
| 10 | T C A G A C G T T C G A T A G C A G T C C G T A A C T G G T A C A C G T A C T G A T C G A G T C | Atoh1(bHLH)/Cerebellum-Atoh1-ChIP-Seq(GSE22111)/Homer | 1e-88 | -2.039e+02 | 0.0000 | 3152.0 | 24.46% | 6358.9 | 17.46% | motif file (matrix) | svg |
| 11 | A T G C G A T C C G A T A C G T A C G T A C T G C A G T A G C T | Sox3(HMG)/NPC-Sox3-ChIP-Seq(GSE33059)/Homer | 1e-81 | -1.881e+02 | 0.0000 | 3815.0 | 29.61% | 8129.3 | 22.32% | motif file (matrix) | svg |
| 12 | A C G T T C G A T C G A A G T C G T C A T A C G A T G C A C G T A C T G A G C T | Myf5(bHLH)/GM-Myf5-ChIP-Seq(GSE24852)/Homer | 1e-79 | -1.839e+02 | 0.0000 | 2164.0 | 16.79% | 4073.0 | 11.18% | motif file (matrix) | svg |
| 13 | A G T C A G T C C G A T A C G T A C G T A C T G A C G T A G C T A G T C A G T C | Sox4(HMG)/proB-Sox4-ChIP-Seq(GSE50066)/Homer | 1e-79 | -1.835e+02 | 0.0000 | 2142.0 | 16.62% | 4023.7 | 11.05% | motif file (matrix) | svg |
| 14 | T G A C A G T C C G T A A C T G G T A C A C G T A C T G A C G T G A C T G A T C | Twist2(bHLH)/Myoblast-Twist2.Ty1-ChIP-Seq(GSE127998)/Homer | 1e-78 | -1.811e+02 | 0.0000 | 4515.0 | 35.04% | 9996.2 | 27.45% | motif file (matrix) | svg |
| 15 | T C G A T C G A A G T C C G T A C T A G T A G C A C G T A C T G | MyoG(bHLH)/C2C12-MyoG-ChIP-Seq(GSE36024)/Homer | 1e-77 | -1.787e+02 | 0.0000 | 3096.0 | 24.03% | 6370.6 | 17.49% | motif file (matrix) | svg |
| 16 | T A C G T G C A A G T C C G T A A C G T T G A C A C G T A C T G A C T G G C A T | TCF4(bHLH)/SHSY5Y-TCF4-ChIP-Seq(GSE96915)/Homer | 1e-67 | -1.565e+02 | 0.0000 | 3907.0 | 30.32% | 8591.6 | 23.59% | motif file (matrix) | svg |
| 17 | T C G A T G A C G T A C C G T A C A G T T G A C A C G T A C T G A G C T A G C T | NeuroG2(bHLH)/Fibroblast-NeuroG2-ChIP-Seq(GSE75910)/Homer | 1e-67 | -1.559e+02 | 0.0000 | 3865.0 | 30.00% | 8486.4 | 23.30% | motif file (matrix) | svg |
| 18 | C T G A T C A G A G T C C G T A A T C G A T G C C G A T A C T G A G T C G A C T A T C G A G T C | MyoD(bHLH)/Myotube-MyoD-ChIP-Seq(GSE21614)/Homer | 1e-66 | -1.536e+02 | 0.0000 | 2461.0 | 19.10% | 4954.3 | 13.60% | motif file (matrix) | svg |
| 19 | T C A G C T A G A T G C G A C T T G C A C G T A A G C T A C G T C T G A T A C G | En1(Homeobox)/SUM149-EN1-ChIP-Seq(GSE120957)/Homer | 1e-62 | -1.429e+02 | 0.0000 | 3502.0 | 27.18% | 7647.0 | 21.00% | motif file (matrix) | svg |
| 20 | A T G C T A C G A G T C G A C T T G C A C T G A G A C T A C G T C T G A T C A G | LHX9(Homeobox)/Hct116-LHX9.V5-ChIP-Seq(GSE116822)/Homer | 1e-60 | -1.397e+02 | 0.0000 | 2755.0 | 21.38% | 5771.1 | 15.85% | motif file (matrix) | svg |
| 21 | A C G T A G T C A G T C C G A T A C G T A C G T A C T G A C G T A T G C G A C T A C T G T A C G | Sox21(HMG)/ESC-SOX21-ChIP-Seq(GSE110505)/Homer | 1e-59 | -1.369e+02 | 0.0000 | 3915.0 | 30.38% | 8765.5 | 24.07% | motif file (matrix) | svg |
| 22 | T G C A A G C T C T G A A T C G G A C T C T A G G T A C G A T C G T C A A G T C G T A C G A C T C T A G A T C G G C A T C A T G C A T G G A T C G T A C C T G A | CTCF(Zf)/CD4+-CTCF-ChIP-Seq(Barski\_et\_al.)/Homer | 1e-58 | -1.355e+02 | 0.0000 | 611.0 | 4.74% | 836.3 | 2.30% | motif file (matrix) | svg |
| 23 | C G T A C A T G A G T C G A C T T G C A C G T A A C G T A C G T C T G A T C A G | Lhx1(Homeobox)/EmbryoCarcinoma-Lhx1-ChIP-Seq(GSE70957)/Homer | 1e-58 | -1.340e+02 | 0.0000 | 2203.0 | 17.10% | 4443.9 | 12.20% | motif file (matrix) | svg |
| 24 | C T A G A G T C T A C G T A C G T G A C C G T A A C T G T A G C G C A T C A T G A T G C A G C T | Ascl1(bHLH)/NeuralTubes-Ascl1-ChIP-Seq(GSE55840)/Homer | 1e-56 | -1.310e+02 | 0.0000 | 4118.0 | 31.96% | 9348.3 | 25.67% | motif file (matrix) | svg |
| 25 | C T G A T C A G G T A C G C T A A C T G T G A C G C A T C A T G | SCL(bHLH)/HPC7-Scl-ChIP-Seq(GSE13511)/Homer | 1e-53 | -1.229e+02 | 0.0000 | 9057.0 | 70.29% | 23252.3 | 63.85% | motif file (matrix) | svg |
| 26 | A G C T G T C A C G T A A C G T A C G T C T G A T C A G A T G C | Lhx2(Homeobox)/HFSC-Lhx2-ChIP-Seq(GSE48068)/Homer | 1e-52 | -1.205e+02 | 0.0000 | 2074.0 | 16.10% | 4212.9 | 11.57% | motif file (matrix) | svg |
| 27 | A T G C A G T C G A T C C G T A A C G T A C G T A C T G A C G T A G C T G A T C | Sox2(HMG)/mES-Sox2-ChIP-Seq(GSE11431)/Homer | 1e-49 | -1.140e+02 | 0.0000 | 2000.0 | 15.52% | 4073.9 | 11.19% | motif file (matrix) | svg |
| 28 | T C G A C A G T A C T G A G C T C G T A C G T A A C G T A C G T C T G A T A G C | Dlx3(Homeobox)/Kerainocytes-Dlx3-ChIP-Seq(GSE89884)/Homer | 1e-49 | -1.131e+02 | 0.0000 | 1249.0 | 9.69% | 2291.2 | 6.29% | motif file (matrix) | svg |
| 29 | A C T G C A G T A C G T C G T A C G T A A C G T A C T G C T G A | Nkx6.1(Homeobox)/Islet-Nkx6.1-ChIP-Seq(GSE40975)/Homer | 1e-46 | -1.077e+02 | 0.0000 | 4425.0 | 34.34% | 10377.4 | 28.49% | motif file (matrix) | svg |
| 30 | T C A G T G A C G T A C C G T A A C G T T G A C A C G T T C A G A G C T G A C T | NeuroD1(bHLH)/Islet-NeuroD1-ChIP-Seq(GSE30298)/Homer | 1e-46 | -1.072e+02 | 0.0000 | 2203.0 | 17.10% | 4619.1 | 12.68% | motif file (matrix) | svg |
| 31 | C G T A C G T A A G C T G A C T T G C A G T C A A C G T A G C T C T G A T C A G | Lhx3(Homeobox)/Neuron-Lhx3-ChIP-Seq(GSE31456)/Homer | 1e-45 | -1.056e+02 | 0.0000 | 3078.0 | 23.89% | 6851.5 | 18.81% | motif file (matrix) | svg |
| 32 | A T G C T A C G A G C T T G C A C G T A C G A T A C G T C T G A | DLX5(Homeobox)/BasalGanglia-Dlx5-ChIP-seq(GSE124936)/Homer | 1e-45 | -1.047e+02 | 0.0000 | 1476.0 | 11.46% | 2866.6 | 7.87% | motif file (matrix) | svg |
| 33 | G A C T C T A G A T G C A G T C G T C A T A C G A T G C A T C G | HIC1(Zf)/Treg-ZBTB29-ChIP-Seq(GSE99889)/Homer | 1e-44 | -1.031e+02 | 0.0000 | 5113.0 | 39.68% | 12282.5 | 33.73% | motif file (matrix) | svg |
| 34 | A T C G A T C G A T G C G A C T T C G A C G T A G C A T A G C T C T G A T A C G | DLX2(Homeobox)/BasalGanglia-Dlx2-ChIP-seq(GSE124936)/Homer | 1e-42 | -9.813e+01 | 0.0000 | 2767.0 | 21.47% | 6114.6 | 16.79% | motif file (matrix) | svg |
| 35 | C T G A T C G A C G T A A T G C C G T A C G T A C G A T C T A G T C A G G A T C | Sox15(HMG)/CPA-Sox15-ChIP-Seq(GSE62909)/Homer | 1e-39 | -8.998e+01 | 0.0000 | 2281.0 | 17.70% | 4945.0 | 13.58% | motif file (matrix) | svg |
| 36 | A T G C G T A C C G T A A G C T G C A T T A C G A G C T A G C T A G T C A G C T | Sox6(HMG)/Myotubes-Sox6-ChIP-Seq(GSE32627)/Homer | 1e-35 | -8.143e+01 | 0.0000 | 3172.0 | 24.62% | 7317.1 | 20.09% | motif file (matrix) | svg |
| 37 | T A G C A G T C T G A C A G T C C T A G A T C G A G T C C A T G T G A C A G T C G T A C A G T C A G T C G C A T C T A G A T C G G C A T A C T G A T C G G A T C | BORIS(Zf)/K562-CTCFL-ChIP-Seq(GSE32465)/Homer | 1e-34 | -8.011e+01 | 0.0000 | 815.0 | 6.33% | 1460.5 | 4.01% | motif file (matrix) | svg |
| 38 | C T G A T G A C T G A C C G T A A C G T T G A C A G C T C T A G A C G T G A C T | Olig2(bHLH)/Neuron-Olig2-ChIP-Seq(GSE30882)/Homer | 1e-34 | -7.881e+01 | 0.0000 | 4333.0 | 33.63% | 10436.1 | 28.66% | motif file (matrix) | svg |
| 39 | A T C G A T G C A G T C T A G C G A C T T C G A G C T A G C A T A G C T C T G A | DLX1(Homeobox)/BasalGanglia-Dlx1-ChIP-seq(GSE124936)/Homer | 1e-33 | -7.726e+01 | 0.0000 | 2415.0 | 18.74% | 5393.0 | 14.81% | motif file (matrix) | svg |
| 40 | T G C A C T G A A T G C G T C A A C G T A T G C A C G T A C T G A C T G T G C A | ZBTB18(Zf)/HEK293-ZBTB18.GFP-ChIP-Seq(GSE58341)/Homer | 1e-32 | -7.506e+01 | 0.0000 | 1365.0 | 10.59% | 2782.5 | 7.64% | motif file (matrix) | svg |
| 41 | G C A T C G T A C G A T A C T G A G T C G C T A C T G A C G T A C A G T A C T G C G T A T C A G | Oct6(POU,Homeobox)/NPC-Pou3f1-ChIP-Seq(GSE35496)/Homer | 1e-31 | -7.311e+01 | 0.0000 | 877.0 | 6.81% | 1636.3 | 4.49% | motif file (matrix) | svg |
| 42 | G A C T C G T A A C G T A C T G A G T C C G T A C T G A C G T A C A G T A C G T G T C A T C A G | Brn1(POU,Homeobox)/NPC-Brn1-ChIP-Seq(GSE35496)/Homer | 1e-30 | -7.129e+01 | 0.0000 | 674.0 | 5.23% | 1184.9 | 3.25% | motif file (matrix) | svg |
| 43 | C T A G T A C G G A T C G T A C G C T A A G C T A G C T G T C A T C G A T A G C | Nanog(Homeobox)/mES-Nanog-ChIP-Seq(GSE11724)/Homer | 1e-29 | -6.794e+01 | 0.0000 | 7356.0 | 57.09% | 18972.9 | 52.10% | motif file (matrix) | svg |
| 44 | T A C G T A C G C T A G T C A G A G T C C G T A A T C G A T G C A C G T A C T G A G T C G A C T | Ascl2(bHLH)/ESC-Ascl2-ChIP-Seq(GSE97712)/Homer | 1e-28 | -6.670e+01 | 0.0000 | 2950.0 | 22.89% | 6887.7 | 18.91% | motif file (matrix) | svg |
| 45 | C A T G C T A G A G C T G A C T C A T G A G T C G A T C G C T A C G A T C T A G T C A G G T A C C T G A T C G A | X-box(HTH)/NPC-H3K4me1-ChIP-Seq(GSE16256)/Homer | 1e-26 | -6.115e+01 | 0.0000 | 359.0 | 2.79% | 545.4 | 1.50% | motif file (matrix) | svg |
| 46 | A G T C A T C G C T A G A G C T G A C T C T A G A G T C A G T C G C T A C A G T T C A G T C A G G A T C C T G A T C G A G A T C | RFX(HTH)/K562-RFX3-ChIP-Seq(SRA012198)/Homer | 1e-25 | -5.975e+01 | 0.0000 | 308.0 | 2.39% | 446.6 | 1.23% | motif file (matrix) | svg |
| 47 | A T G C G A T C C G T A A G C T C A G T A T C G G C A T A G C T G A C T A C T G | Sox17(HMG)/Endoderm-Sox17-ChIP-Seq(GSE61475)/Homer | 1e-25 | -5.782e+01 | 0.0000 | 1449.0 | 11.25% | 3116.1 | 8.56% | motif file (matrix) | svg |
| 48 | A G C T G C A T A C T G A C G T A G T C A C G T C T A G T A C G | Smad3(MAD)/NPC-Smad3-ChIP-Seq(GSE36673)/Homer | 1e-24 | -5.728e+01 | 0.0000 | 6128.0 | 47.56% | 15662.6 | 43.01% | motif file (matrix) | svg |
| 49 | C T A G A G C T G A C T C A T G A G T C A G T C G T C A C A G T C T A G T C A G G T A C C T G A T C G A G A T C T G A C | Rfx2(HTH)/LoVo-RFX2-ChIP-Seq(GSE49402)/Homer | 1e-24 | -5.569e+01 | 0.0000 | 329.0 | 2.55% | 501.2 | 1.38% | motif file (matrix) | svg |
| 50 | A C T G T C A G A G C T G A C T C A T G A G T C A G T C G C T A C G A T C T A G T C A G G T A C C T G A T C G A | Rfx1(HTH)/NPC-H3K4me1-ChIP-Seq(GSE16256)/Homer | 1e-22 | -5.217e+01 | 0.0000 | 578.0 | 4.49% | 1058.3 | 2.91% | motif file (matrix) | svg |
| 51 | T C G A A G T C C G T A A T C G T A G C A C G T A C T G A G C T A C G T A G T C | Ptf1a(bHLH)/Panc1-Ptf1a-ChIP-Seq(GSE47459)/Homer | 1e-20 | -4.807e+01 | 0.0000 | 5797.0 | 44.99% | 14883.0 | 40.87% | motif file (matrix) | svg |
| 52 | A G T C C G A T C T G A C G T A A C G T C A G T T C A G T G A C | Isl1(Homeobox)/Neuron-Isl1-ChIP-Seq(GSE31456)/Homer | 1e-20 | -4.654e+01 | 0.0000 | 3478.0 | 26.99% | 8542.8 | 23.46% | motif file (matrix) | svg |
| 53 | T G A C A T G C C G T A A T C G A T G C C A G T C A T G A C T G A G T C G T A C | HEB(bHLH)/mES-Heb-ChIP-Seq(GSE53233)/Homer | 1e-19 | -4.540e+01 | 0.0000 | 4441.0 | 34.47% | 11176.6 | 30.69% | motif file (matrix) | svg |
| 54 | T C G A A C G T A C G T C T G A G A T C T C A G G A C T G T C A C G T A A G C T G T C A C T A G A G C T A C G T T C G A | NFIL3(bZIP)/HepG2-NFIL3-ChIP-Seq(Encode)/Homer | 1e-19 | -4.455e+01 | 0.0000 | 1011.0 | 7.85% | 2137.6 | 5.87% | motif file (matrix) | svg |
| 55 | C G T A T G A C T C G A A G T C C G T A A T C G A T G C A C G T A C T G A G T C | E2A(bHLH)/proBcell-E2A-ChIP-Seq(GSE21978)/Homer | 1e-18 | -4.275e+01 | 0.0000 | 3552.0 | 27.57% | 8797.3 | 24.16% | motif file (matrix) | svg |
| 56 | A T G C C T G A A T C G T A C G A G T C C G A T T C A G C G A T C T A G A G C T G T C A G T C A C G T A A G T C C G T A T A C G C T G A | Fox:Ebox(Forkhead,bHLH)/Panc1-Foxa2-ChIP-Seq(GSE47459)/Homer | 1e-17 | -4.090e+01 | 0.0000 | 2045.0 | 15.87% | 4809.4 | 13.21% | motif file (matrix) | svg |
| 57 | C G T A A C G T A G C T C G A T C T A G G T A C C G T A A G C T C G T A G C T A | Oct4(POU,Homeobox)/mES-Oct4-ChIP-Seq(GSE11431)/Homer | 1e-17 | -4.011e+01 | 0.0000 | 896.0 | 6.95% | 1889.9 | 5.19% | motif file (matrix) | svg |
| 58 | T A G C G T A C A G T C G T A C C G A T A G T C A G T C A G T C A G T C A G T C C G T A G A T C | Zfp281(Zf)/ES-Zfp281-ChIP-Seq(GSE81042)/Homer | 1e-16 | -3.730e+01 | 0.0000 | 839.0 | 6.51% | 1771.1 | 4.86% | motif file (matrix) | svg |
| 59 | A T G C A T G C A T C G T A C G A G C T A G T C G C T A A G T C T C A G G A C T A C T G T C G A | E-box(bHLH)/Promoter/Homer | 1e-15 | -3.599e+01 | 0.0000 | 218.0 | 1.69% | 337.4 | 0.93% | motif file (matrix) | svg |
| 60 | T G A C C T G A C T A G C T G A C G T A A G T C C T G A A C G T G C A T T A G C G C A T A T C G G A C T G A C T G A T C | GRE(NR),IR3/RAW264.7-GRE-ChIP-Seq(Unpublished)/Homer | 1e-15 | -3.587e+01 | 0.0000 | 520.0 | 4.04% | 1013.9 | 2.78% | motif file (matrix) | svg |
| 61 | T A C G C T G A C T A G T C G A C G T A A G T C C T G A A T C G G C A T T A G C G A C T A C T G A C G T A G C T A G T C G A C T | GRE(NR),IR3/A549-GR-ChIP-Seq(GSE32465)/Homer | 1e-15 | -3.561e+01 | 0.0000 | 319.0 | 2.48% | 555.9 | 1.53% | motif file (matrix) | svg |
| 62 | T C A G A C G T A G T C T C G A A G T C T C A G G C A T C T A G C T A G A G C T | Usf2(bHLH)/C2C12-Usf2-ChIP-Seq(GSE36030)/Homer | 1e-15 | -3.510e+01 | 0.0000 | 661.0 | 5.13% | 1354.9 | 3.72% | motif file (matrix) | svg |
| 63 | C A G T T C A G A G C T G A C T A C G T A G T C G A T C G A C T C T G A A C T G G A T C C G T A C T G A A G T C G T A C | Rfx6(HTH)/Min6b1-Rfx6.HA-ChIP-Seq(GSE62844)/Homer | 1e-14 | -3.426e+01 | 0.0000 | 2544.0 | 19.74% | 6216.6 | 17.07% | motif file (matrix) | svg |
| 64 | A G C T C A T G G C A T G A T C T G C A C T A G G A T C A C G T | Tgif2(Homeobox)/mES-Tgif2-ChIP-Seq(GSE55404)/Homer | 1e-14 | -3.361e+01 | 0.0000 | 5869.0 | 45.55% | 15343.2 | 42.13% | motif file (matrix) | svg |
| 65 | A G T C A C G T A C T G A G C T A C G T A C G T G T C A A G T C | Foxo1(Forkhead)/RAW-Foxo1-ChIP-Seq(Fan\_et\_al.)/Homer | 1e-14 | -3.247e+01 | 0.0000 | 3639.0 | 28.24% | 9200.4 | 25.26% | motif file (matrix) | svg |
| 66 | C G A T C T A G A C G T G T C A C G T A C G T A A G T C C G T A | Foxo3(Forkhead)/U2OS-Foxo3-ChIP-Seq(E-MTAB-2701)/Homer | 1e-13 | -3.164e+01 | 0.0000 | 1265.0 | 9.82% | 2895.5 | 7.95% | motif file (matrix) | svg |
| 67 | G A C T T C G A C G A T T A C G C T A G T A C G A C T G A T G C G T A C G T A C | Zac1(Zf)/Neuro2A-Plagl1-ChIP-Seq(GSE75942)/Homer | 1e-13 | -3.097e+01 | 0.0000 | 5827.0 | 45.22% | 15279.5 | 41.96% | motif file (matrix) | svg |
| 68 | A C T G G T C A A C G T G C A T C G A T T C A G G T A C G T C A A C G T C T G A | Oct11(POU,Homeobox)/NCIH1048-POU2F3-ChIP-seq(GSE115123)/Homer | 1e-13 | -3.053e+01 | 0.0000 | 611.0 | 4.74% | 1266.7 | 3.48% | motif file (matrix) | svg |
| 69 | G C A T C T A G G T A C A G T C C G A T A C T G C T A G C T A G G T A C G C T A | ZNF416(Zf)/HEK293-ZNF416.GFP-ChIP-Seq(GSE58341)/Homer | 1e-13 | -3.021e+01 | 0.0000 | 3177.0 | 24.66% | 7984.5 | 21.92% | motif file (matrix) | svg |
| 70 | C A T G A G T C A G C T C G T A C G A T C G A T G C A T G C A T C G A T C T G A C A T G T G A C | Mef2d(MADS)/Retina-Mef2d-ChIP-Seq(GSE61391)/Homer | 1e-13 | -3.009e+01 | 0.0000 | 320.0 | 2.48% | 583.2 | 1.60% | motif file (matrix) | svg |
| 71 | C G T A A C G T A C T G G T A C C G T A A C G T C G T A C G T A A C G T A C T G A G T C C G T A A C G T C T G A G C A T | OCT:OCT-short(POU,Homeobox)/NPC-OCT6-ChIP-Seq(GSE43916)/Homer | 1e-12 | -2.943e+01 | 0.0000 | 1053.0 | 8.17% | 2377.4 | 6.53% | motif file (matrix) | svg |
| 72 | G C T A C G A T C T A G G T A C G C T A C G A T C G T A G C T A C G A T C A G T G A T C G C T A | Pit1+1bp(Homeobox)/GCrat-Pit1-ChIP-Seq(GSE58009)/Homer | 1e-12 | -2.815e+01 | 0.0000 | 534.0 | 4.14% | 1097.3 | 3.01% | motif file (matrix) | svg |
| 73 | C T A G T A G C A G T C G T C A C T G A A C G T C G T A C G T A C G T A G C T A | Hoxd13(Homeobox)/ChickenMSG-Hoxd13.Flag-ChIP-Seq(GSE86088)/Homer | 1e-11 | -2.738e+01 | 0.0000 | 2637.0 | 20.47% | 6574.7 | 18.05% | motif file (matrix) | svg |
| 74 | C G T A C T G A C T A G C G T A C G T A A G T C C G T A C A G T G C A T G T C A C G A T A C T G A C G T G C A T G A T C | PGR(NR)/EndoStromal-PGR-ChIP-Seq(GSE69539)/Homer | 1e-11 | -2.737e+01 | 0.0000 | 450.0 | 3.49% | 901.3 | 2.47% | motif file (matrix) | svg |
| 75 | G A C T A G T C C G A T A C T G C T G A T G A C G T A C C G T A A T C G G C A T C T G A C T A G | Bcl11a(Zf)/HSPC-BCL11A-ChIP-Seq(GSE104676)/Homer | 1e-11 | -2.709e+01 | 0.0000 | 1479.0 | 11.48% | 3503.2 | 9.62% | motif file (matrix) | svg |
| 76 | T C A G T A C G G A T C G T A C T C G A C G A T C T G A C G T A G C T A C G T A | Hoxd11(Homeobox)/ChickenMSG-Hoxd11.Flag-ChIP-Seq(GSE86088)/Homer | 1e-11 | -2.688e+01 | 0.0000 | 4035.0 | 31.32% | 10390.7 | 28.53% | motif file (matrix) | svg |
| 77 | T C G A A C G T A C T G C T G A A G T C T C A G A G C T G T A C C G T A A G C T G A T C T C G A | JunD(bZIP)/K562-JunD-ChIP-Seq/Homer | 1e-11 | -2.673e+01 | 0.0000 | 215.0 | 1.67% | 365.3 | 1.00% | motif file (matrix) | svg |
| 78 | C T A G T A C G G A T C G T C A T C G A A C G T T C A G C G T A C G T A C G T A | Hoxd10(Homeobox)/ChickenMSG-Hoxd10.Flag-ChIP-Seq(GSE86088)/Homer | 1e-11 | -2.654e+01 | 0.0000 | 2028.0 | 15.74% | 4958.8 | 13.62% | motif file (matrix) | svg |
| 79 | A G T C G C A T C G T A C G T A G T A C A C G T A C T G G A T C G A T C T C G A | BMYB(HTH)/Hela-BMYB-ChIP-Seq(GSE27030)/Homer | 1e-11 | -2.639e+01 | 0.0000 | 2813.0 | 21.83% | 7066.5 | 19.40% | motif file (matrix) | svg |
| 80 | G C T A A C G T C T A G G T C A C G T A A C G T C G T A C G A T C A G T A G T C C G T A A C G T C T A G C T G A A T C G | OCT:OCT(POU,Homeobox)/NPC-Brn1-ChIP-Seq(GSE35496)/Homer | 1e-11 | -2.608e+01 | 0.0000 | 33.0 | 0.26% | 21.4 | 0.06% | motif file (matrix) | svg |
| 81 | G C T A A G C T T A C G G T C A C T G A C G A T C T G A G C A T C A G T A G T C | Brn2(POU,Homeobox)/NPC-Brn2-ChIP-Seq(GSE35496)/Homer | 1e-11 | -2.563e+01 | 0.0000 | 180.0 | 1.40% | 294.4 | 0.81% | motif file (matrix) | svg |
| 82 | C T A G T A C G G A C T T G C A T G C A C G A T T A C G C T G A T C G A C T G A | Hoxa10(Homeobox)/ChickenMSG-Hoxa10.Flag-ChIP-Seq(GSE86088)/Homer | 1e-10 | -2.510e+01 | 0.0000 | 1007.0 | 7.82% | 2307.0 | 6.33% | motif file (matrix) | svg |
| 83 | T G A C T A G C T C A G T C G A T C G A C G T A A G T C C G T A C G T A C G A T C T A G T A C G | Sox7(HMG)/ESC-Sox7-ChIP-Seq(GSE133899)/Homer | 1e-10 | -2.507e+01 | 0.0000 | 624.0 | 4.84% | 1341.5 | 3.68% | motif file (matrix) | svg |
| 84 | T A G C G T A C C G T A C T A G A C T G T G C A C G T A A T G C C G T A A T C G | AR-halfsite(NR)/LNCaP-AR-ChIP-Seq(GSE27824)/Homer | 1e-10 | -2.497e+01 | 0.0000 | 7485.0 | 58.09% | 20096.0 | 55.18% | motif file (matrix) | svg |
| 85 | T C A G G A C T C A G T C T G A A G C T C T A G G A C T T G C A C T G A A G T C | HLF(bZIP)/HSC-HLF.Flag-ChIP-Seq(GSE69817)/Homer | 1e-10 | -2.441e+01 | 0.0000 | 1240.0 | 9.62% | 2915.7 | 8.01% | motif file (matrix) | svg |
| 86 | C A G T C G A T G C A T G C A T G T C A A G C T C A T G C T A G A T G C G T A C | Hoxa11(Homeobox)/ChickenMSG-Hoxa11.Flag-ChIP-Seq(GSE86088)/Homer | 1e-10 | -2.435e+01 | 0.0000 | 3810.0 | 29.57% | 9824.9 | 26.98% | motif file (matrix) | svg |
| 87 | T C A G A G C T A T G C C G T A A G T C T C A G A C G T A T C G T C G A A G T C G A T C T G A C | TFE3(bHLH)/MEF-TFE3-ChIP-Seq(GSE75757)/Homer | 1e-10 | -2.407e+01 | 0.0000 | 208.0 | 1.61% | 361.7 | 0.99% | motif file (matrix) | svg |
| 88 | G T C A A C G T C T A G G T C A C G T A G C A T C G T A C G T A G C A T C A G T A G T C C G T A C A G T C T A G C T G A | OCT:OCT(POU,Homeobox,IR1)/NPC-Brn2-ChIP-Seq(GSE35496)/Homer | 1e-10 | -2.401e+01 | 0.0000 | 53.0 | 0.41% | 52.7 | 0.14% | motif file (matrix) | svg |
| 89 | A G C T T G A C C G A T C G A T C T A G A C G T C A G T C A G T G C T A A G T C | FOXK1(Forkhead)/HEK293-FOXK1-ChIP-Seq(GSE51673)/Homer | 1e-10 | -2.348e+01 | 0.0000 | 1730.0 | 13.43% | 4214.2 | 11.57% | motif file (matrix) | svg |
| 90 | T C G A G C A T A C T G C T G A A G T C T C A G G A C T G T A C C G T A A G C T A G T C G A T C | c-Jun-CRE(bZIP)/K562-cJun-ChIP-Seq(GSE31477)/Homer | 1e-10 | -2.342e+01 | 0.0000 | 545.0 | 4.23% | 1160.4 | 3.19% | motif file (matrix) | svg |
| 91 | G A C T G T A C T G C A A C G T G A T C G C T A T C G A A C G T A G T C C G T A | Pdx1(Homeobox)/Islet-Pdx1-ChIP-Seq(SRA008281)/Homer | 1e-9 | -2.291e+01 | 0.0000 | 1566.0 | 12.15% | 3789.9 | 10.41% | motif file (matrix) | svg |
| 92 | T G A C C T G A C T A G T C G A C T G A A T G C C G T A A C T G G C A T G T A C G C A T A T C G G C A T A G C T G A T C | PR(NR)/T47D-PR-ChIP-Seq(GSE31130)/Homer | 1e-9 | -2.283e+01 | 0.0000 | 4410.0 | 34.23% | 11513.0 | 31.61% | motif file (matrix) | svg |
| 93 | T A G C G A T C G T A C C T G A G C A T C T G A C G T A T G C A C G T A G A T C | Hoxa13(Homeobox)/ChickenMSG-Hoxa13.Flag-ChIP-Seq(GSE86088)/Homer | 1e-9 | -2.273e+01 | 0.0000 | 4065.0 | 31.55% | 10562.0 | 29.00% | motif file (matrix) | svg |
| 94 | T A C G T A G C G C T A C G A T C T A G A C G T C A G T C A G T G C T A A G T C G T C A G C A T | FOXK2(Forkhead)/U2OS-FOXK2-ChIP-Seq(E-MTAB-2204)/Homer | 1e-9 | -2.240e+01 | 0.0000 | 1093.0 | 8.48% | 2559.3 | 7.03% | motif file (matrix) | svg |
| 95 | C T A G A C T G C T A G T C A G T C A G T A C G C T A G A C T G | Maz(Zf)/HepG2-Maz-ChIP-Seq(GSE31477)/Homer | 1e-9 | -2.236e+01 | 0.0000 | 3591.0 | 27.87% | 9265.2 | 25.44% | motif file (matrix) | svg |
| 96 | T A C G T C A G A G C T A T G C C G T A A G T C T C A G A C G T A C T G T C G A | USF1(bHLH)/GM12878-Usf1-ChIP-Seq(GSE32465)/Homer | 1e-9 | -2.218e+01 | 0.0000 | 913.0 | 7.09% | 2098.3 | 5.76% | motif file (matrix) | svg |
| 97 | T A C G C T G A C A T G G A T C G T A C G C A T T C A G T A C G A G C T G T C A G A T C G C A T T A C G C G T A C T A G G A T C G A T C C G A T A C T G T C A G | ZNF322(Zf)/HEK293-ZNF322.GFP-ChIP-Seq(GSE58341)/Homer | 1e-9 | -2.174e+01 | 0.0000 | 764.0 | 5.93% | 1723.1 | 4.73% | motif file (matrix) | svg |
| 98 | C T G A A C T G A C T G A G T C A G T C A G C T C T A G T A C G | ZFX(Zf)/mES-Zfx-ChIP-Seq(GSE11431)/Homer | 1e-9 | -2.154e+01 | 0.0000 | 2922.0 | 22.68% | 7454.8 | 20.47% | motif file (matrix) | svg |
| 99 | T G C A T C G A T C G A A C G T C A T G C G T A A G T C T C A G A C G T G T A C C G T A A G C T | CREB5(bZIP)/LNCaP-CREB5.V5-ChIP-Seq(GSE137775)/Homer | 1e-9 | -2.147e+01 | 0.0000 | 622.0 | 4.83% | 1368.1 | 3.76% | motif file (matrix) | svg |
| 100 | C A T G T A C G T A G C G A T C G A T C A T G C G T A C G A C T T C A G A T G C C G A T A T C G C A G T A C T G G T A C | Zic3(Zf)/mES-Zic3-ChIP-Seq(GSE37889)/Homer | 1e-9 | -2.130e+01 | 0.0000 | 1483.0 | 11.51% | 3594.4 | 9.87% | motif file (matrix) | svg |
| 101 | A G C T A G T C A G T C A C G T C T A G A C G T A C G T A C G T C G T A A G T C G A T C C G T A | FOXP1(Forkhead)/H9-FOXP1-ChIP-Seq(GSE31006)/Homer | 1e-9 | -2.119e+01 | 0.0000 | 783.0 | 6.08% | 1777.6 | 4.88% | motif file (matrix) | svg |
| 102 | C A T G G A C T G C A T A C T G A G C T A C T G A C T G C G T A G C A T A G C T A T C G T A C G | Foxh1(Forkhead)/hESC-FOXH1-ChIP-Seq(GSE29422)/Homer | 1e-9 | -2.115e+01 | 0.0000 | 1024.0 | 7.95% | 2396.8 | 6.58% | motif file (matrix) | svg |
| 103 | A T G C G A C T A C G T C T A G A C G T A C G T A C G T C T G A G A T C G C T A A G C T C G T A | Foxa2(Forkhead)/Liver-Foxa2-ChIP-Seq(GSE25694)/Homer | 1e-8 | -2.016e+01 | 0.0000 | 1395.0 | 10.83% | 3379.9 | 9.28% | motif file (matrix) | svg |
| 104 | G A T C G C A T C T G A C G T A A G C T A G C T C G T A T C G A C T G A A C G T G C A T C G T A | Phox2a(Homeobox)/Neuron-Phox2a-ChIP-Seq(GSE31456)/Homer | 1e-8 | -1.853e+01 | 0.0000 | 549.0 | 4.26% | 1213.5 | 3.33% | motif file (matrix) | svg |
| 105 | C T A G T A C G G A T C G T C A T G C A A C G T T G C A G C T A T C G A T G C A | Hoxa9(Homeobox)/ChickenMSG-Hoxa9.Flag-ChIP-Seq(GSE86088)/Homer | 1e-7 | -1.835e+01 | 0.0000 | 4565.0 | 35.43% | 12051.8 | 33.09% | motif file (matrix) | svg |
| 106 | C A T G G T A C C G T A A G T C C T A G A C G T A C T G G T A C A G T C A G C T | bHLHE40(bHLH)/HepG2-BHLHE40-ChIP-Seq(GSE31477)/Homer | 1e-7 | -1.797e+01 | 0.0000 | 623.0 | 4.84% | 1405.6 | 3.86% | motif file (matrix) | svg |
| 107 | T G C A C G T A G T C A A G C T A G T C G C T A T A G C C G A T C T A G G A T C | Gfi1b(Zf)/HPC7-Gfi1b-ChIP-Seq(GSE22178)/Homer | 1e-7 | -1.717e+01 | 0.0000 | 1270.0 | 9.86% | 3095.9 | 8.50% | motif file (matrix) | svg |
| 108 | A T G C T C A G T C G A G C A T A C T G C G T A A G T C T C A G G A C T T G A C C G T A A G C T | Atf2(bZIP)/3T3L1-Atf2-ChIP-Seq(GSE56872)/Homer | 1e-7 | -1.691e+01 | 0.0000 | 576.0 | 4.47% | 1297.2 | 3.56% | motif file (matrix) | svg |
| 109 | C A T G G T A C G A C T G C T A C G T A C G T A C G T A G C T A G A C T C T G A T C A G G T A C | Mef2c(MADS)/GM12878-Mef2c-ChIP-Seq(GSE32465)/Homer | 1e-7 | -1.680e+01 | 0.0000 | 635.0 | 4.93% | 1449.0 | 3.98% | motif file (matrix) | svg |
| 110 | G T A C G C A T C T G A C G T A G A C T A G C T C A T G T G C A C T G A A C G T G A C T C G T A | Prop1(Homeobox)/GHFT1-PROP1.biotin-ChIP-Seq(GSE77302)/Homer | 1e-7 | -1.677e+01 | 0.0000 | 850.0 | 6.60% | 2001.2 | 5.49% | motif file (matrix) | svg |
| 111 | C T A G A T G C A T G C C G A T A C T G G A C T A T G C G C T A T G A C A G C T T A G C G C T A | PBX1(Homeobox)/MCF7-PBX1-ChIP-Seq(GSE28007)/Homer | 1e-7 | -1.663e+01 | 0.0000 | 192.0 | 1.49% | 361.3 | 0.99% | motif file (matrix) | svg |
| 112 | A T G C A T C G T A C G A G C T A T C G C T G A A G T C C T A G A G C T A T G C C T G A A T G C | CRE(bZIP)/Promoter/Homer | 1e-7 | -1.661e+01 | 0.0000 | 477.0 | 3.70% | 1051.3 | 2.89% | motif file (matrix) | svg |
| 113 | G T A C G A C T C G T A C T G A T C G A C G T A G C T A C A G T C T G A T A C G | Mef2a(MADS)/HL1-Mef2a.biotin-ChIP-Seq(GSE21529)/Homer | 1e-7 | -1.657e+01 | 0.0000 | 623.0 | 4.84% | 1420.7 | 3.90% | motif file (matrix) | svg |
| 114 | T C G A A C T G A C T G C G T A C G T A T C G A A G T C C T G A A T C G G T A C G C A T C A T G | ETS:E-box(ETS,bHLH)/HPC7-Scl-ChIP-Seq(GSE22178)/Homer | 1e-6 | -1.610e+01 | 0.0000 | 208.0 | 1.61% | 401.7 | 1.10% | motif file (matrix) | svg |
| 115 | C G A T A C G T C G T A C G T A A C G T A G C T T G C A C T G A C G T A A C G T A C G T C G T A | Phox2b(Homeobox)/CLBGA-PHOX2B-ChIP-Seq(GSE90683)/Homer | 1e-6 | -1.607e+01 | 0.0000 | 291.0 | 2.26% | 598.1 | 1.64% | motif file (matrix) | svg |
| 116 | T A G C C T A G T C G A G A C T A C T G C T G A A G T C T C A G G C A T T G A C C T G A A G C T | Atf7(bZIP)/3T3L1-Atf7-ChIP-Seq(GSE56872)/Homer | 1e-6 | -1.602e+01 | 0.0000 | 793.0 | 6.15% | 1863.5 | 5.12% | motif file (matrix) | svg |
| 117 | G T C A C T G A T C A G G A T C T G C A T G C A A C G T T C A G C G T A C G T A C G T A G C T A | Hoxd12(Homeobox)/ChickenMSG-Hoxd12.Flag-ChIP-Seq(GSE86088)/Homer | 1e-6 | -1.598e+01 | 0.0000 | 2898.0 | 22.49% | 7511.5 | 20.63% | motif file (matrix) | svg |
| 118 | T C A G A G C T A C G T A C G T G T A C G A T C C G T A C T A G C A T G G T C A C G T A T C G A | STAT4(Stat)/CD4-Stat4-ChIP-Seq(GSE22104)/Homer | 1e-6 | -1.592e+01 | 0.0000 | 1754.0 | 13.61% | 4407.9 | 12.10% | motif file (matrix) | svg |
| 119 | G A C T C A G T A G C T C G A T A G T C G A T C A G T C C G T A A T G C T C A G | Rbpj1(?)/Panc1-Rbpj1-ChIP-Seq(GSE47459)/Homer | 1e-6 | -1.564e+01 | 0.0000 | 2906.0 | 22.55% | 7541.5 | 20.71% | motif file (matrix) | svg |
| 120 | A C T G A G C T A G T C G T C A A G C T T C A G A T G C G A T C G C A T A T C G T C G A T A G C C G A T C A T G T A G C | Pax8(Paired,Homeobox)/Thyroid-Pax8-ChIP-Seq(GSE26938)/Homer | 1e-6 | -1.498e+01 | 0.0000 | 812.0 | 6.30% | 1926.1 | 5.29% | motif file (matrix) | svg |
| 121 | A G T C G A T C G C T A C G A T A C G T T A C G G C A T C T G A G A C T A C T G A G T C G C T A C T G A T C G A C A G T | Oct4:Sox17(POU,Homeobox,HMG)/F9-Sox17-ChIP-Seq(GSE44553)/Homer | 1e-6 | -1.469e+01 | 0.0000 | 226.0 | 1.75% | 452.8 | 1.24% | motif file (matrix) | svg |
| 122 | A T G C A G T C A G C T A G C T A C G T A T C G C G T A C G A T T A G C G A C T | LEF1(HMG)/H1-LEF1-ChIP-Seq(GSE64758)/Homer | 1e-6 | -1.468e+01 | 0.0000 | 1308.0 | 10.15% | 3237.8 | 8.89% | motif file (matrix) | svg |
| 123 | C T G A C T G A C A T G A T C G A G C T A T C G G A C T C A T G C T G A G T C A | Tbr1(T-box)/Cortex-Tbr1-ChIP-Seq(GSE71384)/Homer | 1e-6 | -1.398e+01 | 0.0000 | 2263.0 | 17.56% | 5825.7 | 16.00% | motif file (matrix) | svg |
| 124 | G T C A G C A T G C T A C A G T C T A G G A T C C G T A C T G A C G T A C G A T | Oct2(POU,Homeobox)/Bcell-Oct2-ChIP-Seq(GSE21512)/Homer | 1e-6 | -1.393e+01 | 0.0000 | 513.0 | 3.98% | 1169.6 | 3.21% | motif file (matrix) | svg |
| 125 | C T A G G C A T G A T C C G T A A G T C T C A G G A C T C T A G | CLOCK(bHLH)/Liver-Clock-ChIP-Seq(GSE39860)/Homer | 1e-5 | -1.361e+01 | 0.0000 | 1035.0 | 8.03% | 2531.5 | 6.95% | motif file (matrix) | svg |
| 126 | C A T G A C T G C T A G T C G A T C G A T C G A T C G A T C A G T C A G T C A G T G A C T G A C C G T A A C T G T G C A C G A T A C T G | RBPJ:Ebox(?,bHLH)/Panc1-Rbpj1-ChIP-Seq(GSE47459)/Homer | 1e-5 | -1.352e+01 | 0.0000 | 734.0 | 5.70% | 1743.1 | 4.79% | motif file (matrix) | svg |
| 127 | T G A C G C T A T G A C C G T A T C A G G A T C C G T A C A T G C A T G C T A G C T A G C T A G | Unknown-ESC-element(?)/mES-Nanog-ChIP-Seq(GSE11724)/Homer | 1e-5 | -1.311e+01 | 0.0000 | 1358.0 | 10.54% | 3399.8 | 9.34% | motif file (matrix) | svg |
| 128 | T G A C G T A C C G T A A C T G T G A C C G A T A C T G A T C G A G C T T A C G T C G A T A G C G T A C C G T A A T C G T G A C G C A T A C T G A C T G A T G C | Twist(bHLH)/HMLE-TWIST1-ChIP-Seq(Chang\_et\_al)/Homer | 1e-5 | -1.299e+01 | 0.0000 | 311.0 | 2.41% | 671.7 | 1.84% | motif file (matrix) | svg |
| 129 | C G T A A C G T A C G T A C G T A C G T A G T C A G T C C T G A A G C T A G C T | NFAT(RHD)/Jurkat-NFATC1-ChIP-Seq(Jolma\_et\_al.)/Homer | 1e-5 | -1.272e+01 | 0.0000 | 1544.0 | 11.98% | 3906.1 | 10.73% | motif file (matrix) | svg |
| 130 | G A C T C T A G C T A G A G T C T G C A A C T G A C G T A C G T C T A G T C A G | AMYB(HTH)/Testes-AMYB-ChIP-Seq(GSE44588)/Homer | 1e-5 | -1.261e+01 | 0.0000 | 2903.0 | 22.53% | 7611.7 | 20.90% | motif file (matrix) | svg |
| 131 | T C A G A G C T A T G C C G T A A G C T T C A G C A G T A C T G C T G A A G T C | MITF(bHLH)/MastCells-MITF-ChIP-Seq(GSE48085)/Homer | 1e-5 | -1.234e+01 | 0.0000 | 1720.0 | 13.35% | 4389.3 | 12.05% | motif file (matrix) | svg |
| 132 | T G C A A C T G T A C G A T G C A G T C G A C T T C G A A T C G | ZNF711(Zf)/SHSY5Y-ZNF711-ChIP-Seq(GSE20673)/Homer | 1e-5 | -1.226e+01 | 0.0000 | 4073.0 | 31.61% | 10857.6 | 29.81% | motif file (matrix) | svg |
| 133 | T A G C T A G C G A C T C T A G A G C T A G T C G T C A T G C A A C G T A T G C G C T A T G C A | Pbx3(Homeobox)/GM12878-PBX3-ChIP-Seq(GSE32465)/Homer | 1e-5 | -1.215e+01 | 0.0000 | 507.0 | 3.93% | 1174.2 | 3.22% | motif file (matrix) | svg |
| 134 | T A G C T C A G C A T G G C A T A G C T C G A T A T G C C G T A C G T A G T C A | CHR(?)/Hela-CellCycle-Expression/Homer | 1e-5 | -1.196e+01 | 0.0000 | 1003.0 | 7.78% | 2475.0 | 6.80% | motif file (matrix) | svg |
| 135 | A G T C G A C T A C T G G A T C G T A C C G T A T G A C A G T C C G A T A G C T A C G T A C G T C T A G G A C T C T G A | ZNF7(Zf)/HepG2-ZNF7.Flag-ChIP-Seq(Encode)/Homer | 1e-5 | -1.194e+01 | 0.0000 | 998.0 | 7.75% | 2462.0 | 6.76% | motif file (matrix) | svg |
| 136 | C A T G A C G T A G T C G A T C G A T C G A T C G C T A C T A G C T A G C T A G T C A G T C G A | EBF1(EBF)/Near-E2A-ChIP-Seq(GSE21512)/Homer | 1e-5 | -1.194e+01 | 0.0000 | 2325.0 | 18.04% | 6044.3 | 16.60% | motif file (matrix) | svg |
| 137 | C T A G C T A G A G T C T C A G A C T G A C G T A C G T C T G A | MYB(HTH)/ERMYB-Myb-ChIPSeq(GSE22095)/Homer | 1e-5 | -1.176e+01 | 0.0000 | 3240.0 | 25.15% | 8564.8 | 23.52% | motif file (matrix) | svg |
| 138 | T A C G A T C G G A T C G T C A C T G A G C A T C G A T G C T A T C G A G C T A | Unknown(Homeobox)/Limb-p300-ChIP-Seq/Homer | 1e-5 | -1.159e+01 | 0.0000 | 1051.0 | 8.16% | 2608.0 | 7.16% | motif file (matrix) | svg |
| 139 | G C T A C G T A A G T C A C G T T C G A T A C G A C T G A G C T A G T C T C G A | RORgt(NR)/EL4-RORgt.Flag-ChIP-Seq(GSE56019)/Homer | 1e-5 | -1.155e+01 | 0.0000 | 239.0 | 1.85% | 506.6 | 1.39% | motif file (matrix) | svg |
| 140 | G C T A C G T A A G T C A C G T T C G A T A C G A C T G A G C T A G T C T C G A | RORgt(NR)/EL4-RORgt.Flag-ChIP-Seq(GSE56019)/Homer | 1e-5 | -1.155e+01 | 0.0000 | 239.0 | 1.85% | 506.6 | 1.39% | motif file (matrix) | svg |
| 141 | T A C G A T G C G A C T A C T G A G C T A G T C G T C A T G C A A C G T A G T C G C T A T G C A | Pknox1(Homeobox)/ES-Prep1-ChIP-Seq(GSE63282)/Homer | 1e-5 | -1.152e+01 | 0.0000 | 483.0 | 3.75% | 1120.4 | 3.08% | motif file (matrix) | svg |
| 142 | C T G A C T A G T C G A C G T A A T G C C G T A A T C G C G A T T A G C G C A T A T C G G C A T A G C T G A T C G A C T A G C T | ARE(NR)/LNCAP-AR-ChIP-Seq(GSE27824)/Homer | 1e-4 | -1.125e+01 | 0.0000 | 614.0 | 4.77% | 1462.8 | 4.02% | motif file (matrix) | svg |
| 143 | A T C G T G A C A T G C C T G A T C A G G A C T A G T C C G A T T C A G T C G A C A T G C T A G C T A G C G T A C T A G C T A G C T G A C T A G C T A G A T G C | ZSCAN22(Zf)/HEK293-ZSCAN22.GFP-ChIP-Seq(GSE58341)/Homer | 1e-4 | -1.087e+01 | 0.0001 | 204.0 | 1.58% | 426.9 | 1.17% | motif file (matrix) | svg |
| 144 | C G T A C G T A C G T A A G T C A G C T C T G A A C T G C T A G A G C T A G T C C G T A T C A G | RORg(NR)/Liver-Rorc-ChIP-Seq(GSE101115)/Homer | 1e-4 | -1.068e+01 | 0.0001 | 169.0 | 1.31% | 343.8 | 0.94% | motif file (matrix) | svg |
| 145 | T G A C G T C A A G T C G C T A C T A G A G T C C T G A C A T G A C T G C T A G T A C G T C A G | Zic2(Zf)/ESC-Zic2-ChIP-Seq(SRP197560)/Homer | 1e-4 | -1.066e+01 | 0.0001 | 1119.0 | 8.68% | 2807.7 | 7.71% | motif file (matrix) | svg |
| 146 | G C A T G C A T C T G A A C G T C T G A A C G T C G T A C G T A C G T A A G T C G T C A G T C A | Foxf1(Forkhead)/Lung-Foxf1-ChIP-Seq(GSE77951)/Homer | 1e-4 | -1.054e+01 | 0.0001 | 1461.0 | 11.34% | 3729.9 | 10.24% | motif file (matrix) | svg |
| 147 | G A C T G C A T C T A G C G A T G A T C T C G A C A T G G A T C | Tgif1(Homeobox)/mES-Tgif1-ChIP-Seq(GSE55404)/Homer | 1e-4 | -1.008e+01 | 0.0001 | 5297.0 | 41.11% | 14352.6 | 39.41% | motif file (matrix) | svg |
| 148 | G A C T C G A T C T G A G T C A G A C T C G A T T C G A C G T A G C T A G C T A T G A C G T A C C G T A A C T G T G C A C G A T A C T G A C G T | Pitx1:Ebox(Homeobox,bHLH)/Hindlimb-Pitx1-ChIP-Seq(GSE41591)/Homer | 1e-4 | -9.646e+00 | 0.0002 | 306.0 | 2.37% | 690.6 | 1.90% | motif file (matrix) | svg |
| 149 | T A C G T C G A G A C T A C T G C T G A A G T C T C A G G A C T T G A C C T G A | Atf1(bZIP)/K562-ATF1-ChIP-Seq(GSE31477)/Homer | 1e-3 | -9.177e+00 | 0.0003 | 1075.0 | 8.34% | 2719.5 | 7.47% | motif file (matrix) | svg |
| 150 | A T G C T C G A A G T C A G C T A C G T G T A C A G T C G C T A C T A G C A T G G T C A C T G A T C A G A G T C | Stat3+il21(Stat)/CD4-Stat3-ChIP-Seq(GSE19198)/Homer | 1e-3 | -8.762e+00 | 0.0005 | 1399.0 | 10.86% | 3603.7 | 9.90% | motif file (matrix) | svg |
| 151 | G A C T C T A G C T A G C T A G A C T G T C G A C T G A C T A G C T A G C T A G G T A C G T C A | ZNF467(Zf)/HEK293-ZNF467.GFP-ChIP-Seq(GSE58341)/Homer | 1e-3 | -8.635e+00 | 0.0005 | 2168.0 | 16.83% | 5706.0 | 15.67% | motif file (matrix) | svg |
| 152 | T G C A A G C T A C G T C T A G G A T C C T A G G A T C G T C A C T G A A G T C | CEBP(bZIP)/ThioMac-CEBPb-ChIP-Seq(GSE21512)/Homer | 1e-3 | -8.493e+00 | 0.0006 | 959.0 | 7.44% | 2422.1 | 6.65% | motif file (matrix) | svg |
| 153 | C G A T C T A G A C G T A C G T A C G T C G T A A G C T C G A T A G C T C G T A C T A G T A G C | FoxD3(forkhead)/ZebrafishEmbryo-Foxd3.biotin-ChIP-seq(GSE106676)/Homer | 1e-3 | -8.426e+00 | 0.0006 | 1535.0 | 11.91% | 3981.8 | 10.93% | motif file (matrix) | svg |
| 154 | T A C G A T C G T A G C G A T C A C T G A C G T A G T C A C G T C T A G A T C G | Smad4(MAD)/ESC-SMAD4-ChIP-Seq(GSE29422)/Homer | 1e-3 | -8.405e+00 | 0.0006 | 3503.0 | 27.19% | 9404.4 | 25.82% | motif file (matrix) | svg |
| 155 | G C T A A C G T C T A G G T A C G C T A G A C T C T G A G C A T C A T G G A T C | Pit1(Homeobox)/GCrat-Pit1-ChIP-Seq(GSE58009)/Homer | 1e-3 | -8.226e+00 | 0.0008 | 1500.0 | 11.64% | 3891.2 | 10.68% | motif file (matrix) | svg |
| 156 | G C T A G C A T G A C T G C A T T C A G G T A C G C T A G C A T C T G A G C T A T A G C G C T A C T G A C G A T C T A G | OCT4-SOX2-TCF-NANOG(POU,Homeobox,HMG)/mES-Oct4-ChIP-Seq(GSE11431)/Homer | 1e-3 | -8.192e+00 | 0.0008 | 299.0 | 2.32% | 688.5 | 1.89% | motif file (matrix) | svg |
| 157 | G C T A T C G A C G T A C T A G A G C T G T C A G T C A C G T A A G T C C G T A | FOXA1(Forkhead)/LNCAP-FOXA1-ChIP-Seq(GSE27824)/Homer | 1e-3 | -8.149e+00 | 0.0008 | 2065.0 | 16.03% | 5438.9 | 14.93% | motif file (matrix) | svg |
| 158 | C T G A T C A G C A G T C T A G A C T G C T A G G A T C A T C G A C T G C T G A T C A G G A T C | Sp5(Zf)/mES-Sp5.Flag-ChIP-Seq(GSE72989)/Homer | 1e-3 | -8.110e+00 | 0.0008 | 2669.0 | 20.71% | 7103.3 | 19.50% | motif file (matrix) | svg |
| 159 | A T G C A G T C C T G A A G T C C G A T A C G T A G T C A G T C A C G T A T C G G A C T A C G T | Etv2(ETS)/ES-ER71-ChIP-Seq(GSE59402)/Homer | 1e-3 | -8.109e+00 | 0.0008 | 1918.0 | 14.89% | 5036.7 | 13.83% | motif file (matrix) | svg |
| 160 | A G T C C T G A A G T C C G A T C A G T G A T C A T G C A C T G A T C G G A C T | Fli1(ETS)/CD8-FLI-ChIP-Seq(GSE20898)/Homer | 1e-3 | -7.967e+00 | 0.0010 | 2373.0 | 18.42% | 6291.7 | 17.28% | motif file (matrix) | svg |
| 161 | C T G A C T A G A T C G G C A T A C T G G T A C A T G C C G T A A C T G G C T A A G T C C G T A | Tbox:Smad(T-box,MAD)/ESCd5-Smad2\_3-ChIP-Seq(GSE29422)/Homer | 1e-3 | -7.755e+00 | 0.0012 | 375.0 | 2.91% | 889.1 | 2.44% | motif file (matrix) | svg |
| 162 | G A T C G C A T A C T G C T A G C T G A A G C T G C T A G C T A C T G A T C A G G C A T T G C A A C G T A C G T G A T C G A C T G C A T C T A G T A C G G A C T C T A G C A T G C T A G G T A C T C G A | ZNF136(Zf)/HEK293-ZNF136.GFP-ChIP-Seq(GSE58341)/Homer | 1e-3 | -7.672e+00 | 0.0013 | 152.0 | 1.18% | 324.4 | 0.89% | motif file (matrix) | svg |
| 163 | C G A T C T A G T C G A A G C T C G A T C T G A C G T A A G C T A C T G C T A G A T G C G A T C | Hoxb4(Homeobox)/ES-Hoxb4-ChIP-Seq(GSE34014)/Homer | 1e-3 | -7.661e+00 | 0.0013 | 344.0 | 2.67% | 810.5 | 2.23% | motif file (matrix) | svg |
| 164 | T G A C T C G A C T G A C T G A A T G C G A T C C T A G T A C G G A C T G A C T G A T C T C G A C T G A C T G A A T G C G A T C C T A G A T C G G A C T G A C T | Tcfcp2l1(CP2)/mES-Tcfcp2l1-ChIP-Seq(GSE11431)/Homer | 1e-3 | -7.334e+00 | 0.0018 | 352.0 | 2.73% | 835.7 | 2.29% | motif file (matrix) | svg |
| 165 | A G T C G A C T A G C T C G A T A T C G G C T A C G A T A T C G C G A T A C T G T A C G A C G T | Tcf7(HMG)/GM12878-TCF7-ChIP-Seq(Encode)/Homer | 1e-3 | -7.317e+00 | 0.0018 | 602.0 | 4.67% | 1492.0 | 4.10% | motif file (matrix) | svg |
| 166 | A C G T T G C A A G C T G A T C C T A G C T G A A G C T G T C A T C G A C G T A | CUX1(Homeobox)/K562-CUX1-ChIP-Seq(GSE92882)/Homer | 1e-3 | -7.258e+00 | 0.0019 | 975.0 | 7.57% | 2492.5 | 6.84% | motif file (matrix) | svg |
| 167 | G C A T C T G A T G C A G A C T A G T C G T A C T C A G C G T A A G C T A C G T G C T A G T A C | Pax7(Paired,Homeobox),long/Myoblast-Pax7-ChIP-Seq(GSE25064)/Homer | 1e-3 | -7.255e+00 | 0.0019 | 34.0 | 0.26% | 52.3 | 0.14% | motif file (matrix) | svg |
| 168 | T C G A T A G C T G A C C T G A A G T C A C T G G A C T C A T G | c-Myc(bHLH)/LNCAP-cMyc-ChIP-Seq(Unpublished)/Homer | 1e-3 | -7.239e+00 | 0.0019 | 937.0 | 7.27% | 2390.4 | 6.56% | motif file (matrix) | svg |
| 169 | G T A C G A T C C A G T A G T C A G T C A G T C T G C A G A T C C T G A A T G C G T C A A C G T | WT1(Zf)/Kidney-WT1-ChIP-Seq(GSE90016)/Homer | 1e-2 | -6.894e+00 | 0.0026 | 1496.0 | 11.61% | 3917.5 | 10.76% | motif file (matrix) | svg |
| 170 | T A G C G C A T A G T C G A T C A T G C G A C T C T A G A C T G A C T G C T G A A C T G C T A G A G T C T G A C C G A T | GLIS3(Zf)/Thyroid-Glis3.GFP-ChIP-Seq(GSE103297)/Homer | 1e-2 | -6.886e+00 | 0.0026 | 3220.0 | 24.99% | 8676.3 | 23.82% | motif file (matrix) | svg |
| 171 | A T G C G A C T A C T G C A G T G A T C A C G T T A C G T A C G | Smad2(MAD)/ES-SMAD2-ChIP-Seq(GSE29422)/Homer | 1e-2 | -6.801e+00 | 0.0029 | 3448.0 | 26.76% | 9314.6 | 25.58% | motif file (matrix) | svg |
| 172 | A T C G A G T C A G T C G A C T A T G C C T G A C T A G A C T G T A C G G T A C C T G A C G A T | AP-2gamma(AP2)/MCF7-TFAP2C-ChIP-Seq(GSE21234)/Homer | 1e-2 | -6.758e+00 | 0.0030 | 2491.0 | 19.33% | 6659.0 | 18.28% | motif file (matrix) | svg |
| 173 | A T G C G A T C G A C T A G C T C G A T C G A T G T C A C G A T T C G A A T C G T A G C T A G C | TATA-Box(TBP)/Promoter/Homer | 1e-2 | -6.683e+00 | 0.0032 | 2249.0 | 17.45% | 5993.8 | 16.46% | motif file (matrix) | svg |
| 174 | G C A T C G T A C G A T G A C T A C T G C T G A G A C T G A T C | Hnf6b(Homeobox)/LNCaP-Hnf6b-ChIP-Seq(GSE106305)/Homer | 1e-2 | -6.670e+00 | 0.0032 | 1269.0 | 9.85% | 3304.2 | 9.07% | motif file (matrix) | svg |
| 175 | C T A G C A G T C G T A A C G T A G T C A C T G C G T A A G C T A G T C G A T C | HNF6(Homeobox)/Liver-Hnf6-ChIP-Seq(ERP000394)/Homer | 1e-2 | -6.667e+00 | 0.0032 | 800.0 | 6.21% | 2034.9 | 5.59% | motif file (matrix) | svg |
| 176 | C A T G A G T C G A C T C G T A C G A T G C A T G A C T G C A T C G A T C T A G C A T G T G A C | Mef2b(MADS)/HEK293-Mef2b.V5-ChIP-Seq(GSE67450)/Homer | 1e-2 | -6.637e+00 | 0.0033 | 1211.0 | 9.40% | 3148.0 | 8.64% | motif file (matrix) | svg |
| 177 | T G C A A G C T C A T G C G T A A G C T A C T G G A T C G T C A C G T A A G C T | Atf4(bZIP)/MEF-Atf4-ChIP-Seq(GSE35681)/Homer | 1e-2 | -6.600e+00 | 0.0034 | 394.0 | 3.06% | 955.8 | 2.62% | motif file (matrix) | svg |
| 178 | T C G A C T A G A G T C A G T C C G T A C G T A A C G T T A G C T C A G T A C G | NFY(CCAAT)/Promoter/Homer | 1e-2 | -6.479e+00 | 0.0038 | 1655.0 | 12.84% | 4365.1 | 11.99% | motif file (matrix) | svg |
| 179 | C G A T C T A G C T G A A T G C C T G A T C G A C G T A C T G A T C G A T A G C A G T C C G T A A C T G T C G A A T G C | Hand2(bHLH)/Mesoderm-Hand2-ChIP-Seq(GSE61475)/Homer | 1e-2 | -6.450e+00 | 0.0039 | 976.0 | 7.57% | 2514.5 | 6.90% | motif file (matrix) | svg |
| 180 | G A C T C A G T G A T C G A T C A C G T G A T C C T G A T A C G C G T A G T C A | STAT6(Stat)/Macrophage-Stat6-ChIP-Seq(GSE38377)/Homer | 1e-2 | -6.356e+00 | 0.0042 | 943.0 | 7.32% | 2427.0 | 6.66% | motif file (matrix) | svg |
| 181 | C T A G T C G A C G A T C T A G G C A T C A G T C T A G G A T C C G T A G T C A | CEBP:AP1(bZIP)/ThioMac-CEBPb-ChIP-Seq(GSE21512)/Homer | 1e-2 | -6.295e+00 | 0.0045 | 1144.0 | 8.88% | 2974.5 | 8.17% | motif file (matrix) | svg |
| 182 | A G T C G A C T C A G T G T A C A G T C A T C G T C A G A C T G G T C A C G T A | Stat3(Stat)/mES-Stat3-ChIP-Seq(GSE11431)/Homer | 1e-2 | -6.174e+00 | 0.0050 | 1000.0 | 7.76% | 2586.8 | 7.10% | motif file (matrix) | svg |
| 183 | T G C A C T A G C T A G C T G A C A T G A C T G T G C A G A T C G T C A T G C A G T C A G T C A A G C T C T A G G C A T | ZNF675(Zf)/HEK293-ZNF675.GFP-ChIP-Seq(GSE58341)/Homer | 1e-2 | -6.154e+00 | 0.0051 | 256.0 | 1.99% | 602.1 | 1.65% | motif file (matrix) | svg |
| 184 | C G T A G C T A C G A T C T A G A C G T G T C A C G T A C G T A A G T C C G T A T G C A T A C G | FoxL2(Forkhead)/Ovary-FoxL2-ChIP-Seq(GSE60858)/Homer | 1e-2 | -6.048e+00 | 0.0056 | 1290.0 | 10.01% | 3379.8 | 9.28% | motif file (matrix) | svg |
| 185 | G C T A A C T G T C G A C T G A C T G A A C G T T A G C C T G A C G T A C G A T | Cux2(Homeobox)/Liver-Cux2-ChIP-Seq(GSE35985)/Homer | 1e-2 | -6.022e+00 | 0.0058 | 641.0 | 4.97% | 1621.7 | 4.45% | motif file (matrix) | svg |
| 186 | T C G A T A G C T G C A A C T G A C T G C G T A C G T A C T A G G A C T T A C G | ETS1(ETS)/Jurkat-ETS1-ChIP-Seq(GSE17954)/Homer | 1e-2 | -5.832e+00 | 0.0069 | 2158.0 | 16.75% | 5773.5 | 15.85% | motif file (matrix) | svg |
| 187 | T C G A T A G C G T C A A C T G A C T G C G T A C G T A C T A G A G C T T C A G | ERG(ETS)/VCaP-ERG-ChIP-Seq(GSE14097)/Homer | 1e-2 | -5.744e+00 | 0.0075 | 3223.0 | 25.01% | 8733.4 | 23.98% | motif file (matrix) | svg |
| 188 | C T A G A T C G G A C T A G T C A G T C A G T C G A C T C T G A A C T G C T A G A C T G C T G A | EBF(EBF)/proBcell-EBF-ChIP-Seq(GSE21978)/Homer | 1e-2 | -5.709e+00 | 0.0078 | 463.0 | 3.59% | 1152.1 | 3.16% | motif file (matrix) | svg |
| 189 | C T A G T C G A C T G A C G T A T A C G G A C T T C A G T C G A G T C A T G C A T A C G A G C T | IRF2(IRF)/Erythroblas-IRF2-ChIP-Seq(GSE36985)/Homer | 1e-2 | -5.703e+00 | 0.0078 | 192.0 | 1.49% | 443.2 | 1.22% | motif file (matrix) | svg |
| 190 | T C G A G A C T A G C T T G A C A G C T G T A C T C A G G A T C A T C G T G C A A C T G C T G A | GFX(?)/Promoter/Homer | 1e-2 | -5.602e+00 | 0.0085 | 40.0 | 0.31% | 71.9 | 0.20% | motif file (matrix) | svg |
| 191 | C G A T G A C T C G A T T C A G G A C T A C G T C A G T C T G A G A C T G A C T A G C T C G A T A C T G A T C G G T A C G C T A | NF1:FOXA1(CTF,Forkhead)/LNCAP-FOXA1-ChIP-Seq(GSE27824)/Homer | 1e-2 | -5.589e+00 | 0.0086 | 115.0 | 0.89% | 250.2 | 0.69% | motif file (matrix) | svg |
| 192 | C G T A C G T A C G T A A G T C A G C T C T G A A C T G A C T G A G C T A G T C C G T A C T A G C T A G C T A G T G C A | RORa(NR)/Liver-Rora-ChIP-Seq(GSE101115)/Homer | 1e-2 | -5.571e+00 | 0.0087 | 250.0 | 1.94% | 594.8 | 1.63% | motif file (matrix) | svg |
| 193 | T C A G T A C G T A G C A C G T A C T G C G A T A G T C C G T A T A C G A G T C | Meis1(Homeobox)/MastCells-Meis1-ChIP-Seq(GSE48085)/Homer | 1e-2 | -5.556e+00 | 0.0088 | 3235.0 | 25.11% | 8776.0 | 24.10% | motif file (matrix) | svg |
| 194 | C T G A A G C T A C G T A C G T A G T C G A C T G A C T C T G A C T G A C T A G C G T A C G T A | STAT6(Stat)/CD4-Stat6-ChIP-Seq(GSE22104)/Homer | 1e-2 | -5.468e+00 | 0.0096 | 893.0 | 6.93% | 2314.4 | 6.35% | motif file (matrix) | svg |
| 195 | C A G T G A C T A G T C G C T A C T G A G T C A C G T A A G C T G T C A C T G A C T G A C T G A A C T G A G C T G A T C | MYNN(Zf)/HEK293-MYNN.eGFP-ChIP-Seq(Encode)/Homer | 1e-2 | -5.379e+00 | 0.0104 | 385.0 | 2.99% | 951.5 | 2.61% | motif file (matrix) | svg |
| 196 | T C A G A T C G G A C T A C T G G A C T C A G T C T A G C G T A G T A C C G T A C T A G A T C G | Tbx20(T-box)/Heart-Tbx20-ChIP-Seq(GSE29636)/Homer | 1e-2 | -5.369e+00 | 0.0105 | 458.0 | 3.55% | 1145.6 | 3.15% | motif file (matrix) | svg |
| 197 | T C A G T C A G G C T A C G T A T A C G G A C T T C A G T C G A C T G A C G T A T A C G G A C T | IRF8(IRF)/BMDM-IRF8-ChIP-Seq(GSE77884)/Homer | 1e-2 | -5.206e+00 | 0.0123 | 560.0 | 4.35% | 1421.4 | 3.90% | motif file (matrix) | svg |
| 198 | C T G A A T C G A G C T A G C T A C G T T A G C C T G A T A C G C G A T A C G T G A C T A G T C | ISRE(IRF)/ThioMac-LPS-Expression(GSE23622)/Homer | 1e-2 | -5.168e+00 | 0.0127 | 97.0 | 0.75% | 209.9 | 0.58% | motif file (matrix) | svg |
| 199 | T C A G G T C A G C T A A G T C C G T A A T C G T C G A T C A G C G T A A C T G A C T G A C T G | ZNF768(Zf)/Rajj-ZNF768-ChIP-Seq(GSE111879)/Homer | 1e-2 | -4.976e+00 | 0.0153 | 85.0 | 0.66% | 181.8 | 0.50% | motif file (matrix) | svg |
| 200 | C T G A A C G T A C G T A C G T A G T C G A C T C G A T C T G A A C T G C G T A C G T A T C G A | STAT5(Stat)/mCD4+-Stat5-ChIP-Seq(GSE12346)/Homer | 1e-2 | -4.954e+00 | 0.0155 | 620.0 | 4.81% | 1588.8 | 4.36% | motif file (matrix) | svg |
| 201 | A C T G C A T G T C G A A C G T A C T G C G T A A T C G A C G T G T A C C G T A G A C T A G T C | Fos(bZIP)/TSC-Fos-ChIP-Seq(GSE110950)/Homer | 1e-2 | -4.815e+00 | 0.0177 | 849.0 | 6.59% | 2212.8 | 6.08% | motif file (matrix) | svg |
